# Protocol for a Prospective (P) study to develop a model to stratify the risk (RI) of medication (M) related harm in hospitalized elderly (E) patients in the UK (The PRIME study)

**DOI:** 10.1186/s12877-016-0191-8

**Published:** 2016-01-19

**Authors:** Jennifer Stevenson, Nikesh Parekh, Khalid Ali, Jean Timeyin, Stephen Bremner, Tischa Van Der Cammen, Jane Allen, Rebekah Schiff, Jatinder Harchowal, Graham Davies, Chakravarthi Rajkumar

**Affiliations:** Guy’s and St Thomas‘NHS Foundation Trust, London, UK; Brighton and Sussex Medical School, Brighton, UK; Brighton and Sussex University Hospitals’ Trust, Brighton, UK; Delft University of Technology, Delft, Netherlands; The Royal Marsden NHS Foundation Trust, London, UK; King’s College London, London, UK

**Keywords:** Elderly, Medication-related harm, Risk prediction, Prognostic research, Public health

## Abstract

**Background:**

Medication related harm (MRH) is a common cause of morbidity and hospital admission in the elderly, and has significant cost implications for both primary and secondary healthcare resources. The development of risk prediction models has become an increasingly common phenomenon in medicine and can be useful to guide objective clinical decision making, resource allocation and intervention. There are no risk prediction models that are widely used in clinical practice to identify elderly patients at high risk of MRH following hospital discharge. The aim of this study is to develop a risk prediction model (RPM) to identify elderly patients at high risk of MRH upon discharge from hospital, and to compare this with routine clinical judgment.

**Methods/Design:**

This is a multi-centre, prospective observational study following a cohort of patients for 8 weeks after hospital discharge. Data collection including patient characteristics, medication use, social factors and frailty will take place prior to patient discharge and then the patient will be followed up in the community over the next 8 weeks to determine if they have experienced MRH. Research pharmacists will determine whether patients have experienced MRH by prospectively reviewing records for unplanned emergency department attendance, hospital readmission and GP consultation related to MRH. Research pharmacists will also telephone patients directly to determine self-reported MRH, which patients may not have sought further medical attention for. The data collected will inform the development of a RPM which will be externally validated in a follow-up study.

**Discussion:**

There are no RPMs that are used in clinical practice to help stratify elderly patients at high risk of MRH in the community following hospital discharge, despite this being a significant public health problem. This study plans to develop a clinically useful RPM that is better than routine clinical judgment. As this is a multi-centre study involving clinical settings that serve elderly people of heterogeneous sociodemographic background, it is anticipated that this RPM will be generalizable.

**Electronic supplementary material:**

The online version of this article (doi:10.1186/s12877-016-0191-8) contains supplementary material, which is available to authorized users.

## Background

Medication related harm (MRH) in older people is a significant cause of increased morbidity, hospitalisation, longer hospital stay and increased healthcare costs [1]. In people aged 65 years or older, hospital admissions attributable to medication related problems range between 5.3 to 30.7 % [1]. Adverse Drug Reactions (ADR) is a subset of medication related harm. A large prospective UK study of 18,820 patients reported a prevalence of hospital admission secondary to ADR of 6.5 %, of which almost three-quarters (72 %) were potentially avoidable [2]. Although this study considered all patients aged over 16, the average age of patients admitted with ADR was 76 years old. A more recent UK study of a hospitalized population of very elderly patients (over 80 years of age) reported an inpatient ADR incidence of 13.2 %, with 63 % considered preventable [3]. The annual cost to the NHS of admissions secondary to ADR in the UK was estimated at £466 million [2].

A study in the Netherlands of 106 medical inpatients aged 70 and over found a prevalence of severe ADR of 24 and 12 % of these elderly patients were admitted due to ADR [4].

A large, retrospective US study found that almost 100,000 elderly patients required emergency hospitalization as a direct result of ADR each year, with nearly half occurring in those over the age of 80 years [5]. A meta-analysis highlighted that the elderly were four times more likely to be admitted to hospital as a result of an ADR when compared to younger patients (16.6 % compared to 4.1 %), highlighting the increased vulnerability of an older population [6]. This increased vulnerability of the elderly population is multifactorial and includes polypharmacy, co-morbidities, renal and hepatic impairment and changes in pharmacokinetics and pharmacodynamics, cognitive impairment and altered adherence [7].

Risk prediction models (RPM) are increasingly used within healthcare to personalize and target clinical interventions. Four RPMs have been developed to date to predict ADR in older adults, although none are in current clinical use [8–11]. These models allocate scores to specific clinical parameters to identify patients at high risk of ADR during hospital admission. These four models showed at best a moderate predictive ability (Area under the receiver operator curve 0.623 to 0.73), and have not demonstrated an improvement in routine care. Three of these studies were based on prospective cohort data [9–11], whilst Onder et al’s (2010) GerontoNet ADR risk score was based on retrospective data.

McElnay et al. [10] developed the first tool based on data collected from inpatients at one UK hospital. This study identified several important risk factors for adverse drug events but the model developed had low sensitivity and specificity (40.5 and 69 % respectively). Trivalle et al. [11] developed a risk prediction model based on data collected from several rehabilitation hospital units in Paris, France, that was entirely based on medications as risk variables (number of medications, use of anticoagulant, use of neuroleptic). The other two ADR risk prediction models were based on data collected from a multi-centre European study (GerontoNet) and a study at one large teaching hospital in the UK (Brighton Adverse Drug Reactions Risk Model; BADRI). Both of these models (GerontoNet and BADRI) were developed to identify patients at risk of ADR whilst in hospital, and neither considered social measures as potential risk variables.

Given that at least one-fifth of hospital readmissions of elderly people in the UK are secondary to ADR [12], it is of both ethical and economic importance that elderly patients at high risk of MRH following hospital discharge are identified through objective and evidence based means. Following this process, appropriate mechanisms to reduce the risk of MRH can be initiated. The PRIME study intends to bridge this gap in prognostic research by developing a RPM to identify elderly patients at high risk of MRH at the point of hospital discharge. To our knowledge such a study has not been previously conducted. Prognostic research has thus far lacked transparency and this protocol paper is a step to addressing this issue [13]. If this RPM demonstrates a high prognostic value then it could be incorporated into local care pathways to minimize the occurrence of post-discharge MRH among elderly people. This proposal follows Medical Research Council (MRC) guidance relating to ‘Developing & Evaluating Complex Interventions’ which specifies the need for developmental work prior to a full evaluation [14].

### Study hypothesis

The use of a risk prediction model (RPM) to identify patients at risk of experiencing MRH will better predict these events compared to routine clinical judgment.

### Primary objective

To develop a RPM to identify elderly patients at risk of MRH in the 8 week period following hospital discharge.

### Secondary objectives

1. To compare the predictive power of the RPM to intuitive decisions (i.e. standard care) in relation to actual MRH experienced in the 8 week period following hospital discharge.
2. To determine the frequency of health care utilization secondary to MRH in the 8 week period following hospital discharge.
3. To describe the medications commonly causing MRH, the type of events occurring, their severity and preventability.
4. To describe local factors, such as ethnicity and complexity of health care systems, which impact on MRH frequency, type and severity.

## Methods/Design

The PRIME study is a prospective observational study that aims to develop a risk prediction model (RPM) that (1) can identify elderly patients at high risk of MRH upon discharge from hospital and (2) is superior to routine clinical judgment.

The PRIME study protocol was approved by the National Research Ethics Service, East of England (Norfolk; REC Reference 13/EE/0075), and was funded by the National Institute of Health Research (NIHR)- Research for Patient Benefit (RfPB) (PB-PG-0711-25094) and adopted as a Clinical Research Network portfolio study (Ageing and Primary Care).

Stage I of the study will involve a critical review of the medication RPMs in published literature, as they relate to elderly people, to inform the data collection for Stage II.

**Fig. 1:**
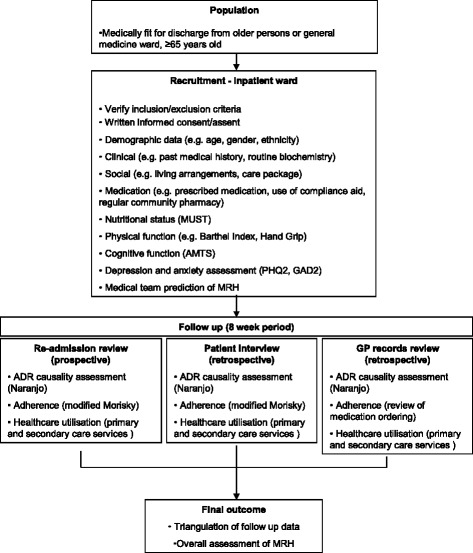
Data collection flow chart. This flow chart outlines the process that the research nurses at respective sites will use to recruit patients for the study from inpatient hosptial wards and the biopsychosocial data that was collected at baseline. The flow chart further outlines the three types of patient follow-up that will be conducted in order to determine medication harm eight weeks following hospital discharge. This includes review of any hospital re-admissions, a patient interview and review of General Practice records. These three sources of information will finally be triangulated to determine overall if medication harm has occurred

Stage II will comprise the development of a new RPM by using a prospective, observational study design to follow a cohort of 1500 elderly patients for 8 weeks after discharge from an acute care setting into the community. Patients will be invited to participate in this study during their inpatient stay and consent (or assent) obtained as close to discharge as possible. The study will collect a range of baseline clinical, medication and social data by trained research nurses that will be potential predictor variables to inform the RPM (Please see Fig. 1 and Additional file 1: Table S2). In addition, the views of the discharging medical team on the likelihood of the patient experiencing MRH will be recorded. During the 8 week follow-up MRH will be determined by a research pharmacist through patient/carer self-report via telephone interview, review of primary care records and assessment of any re-admissions to the recruiting hospital.

Along with using statistical methods to identify predictor variables from the data collected to develop the risk prediction model, an expert panel will be established to identify important predictor variables for inclusion in the model development.

### Setting

This study will be led by the Academic Department of Geriatrics (Brighton & Sussex University Hospitals NHS Trust) in collaboration with the Department of Ageing and Health (Guy’s & St Thomas’ NHS Foundation Trust). The study will be undertaken in acute elderly care inpatient wards at 5 NHS hospitals in the UK. Access to patient primary care information, during the 8-week follow up period, will be facilitated through the UK Primary Care Research Network.

### Population

Patients aged 65 years and over who are judged clinically fit for discharge from the acute Care of the Elderly and General Medicine wards will be eligible to participate. Written consent will be obtained from all participants. Where a patient lacks capacity to consent, their next of kin will be asked to act as a personal consultee and to support their relative taking part in the study. It is important to include those who lack capacity, as we do not wish to exclude those who are most likely to experience MRH i.e. those most vulnerable due to frailty and/or cognitive limitation. If a potential participant lacks capacity and the next of kin is not available, they will not be included in the study. Patients who consent to be included in the study will be allocated a Unique Patient Identifier Number (UPIN).

### Inclusion criteria

Patients must be over the age of 65 years at the time of recruitment and registered with a General Practitioner within the areas covered by the recruiting hospitals.

### Exclusion criteria

- Patients who lack capacity and have no nominated consultee,
- Patients that are transferred to other acute healthcare trusts (but excluding step down or intermediate care facilities),
- Patients who have a short life expectancy, due to a terminal illness

### Definitions

Medication related harm (MRH) for this study will include adverse drug reactions and a failure to receive medication, either following non-adherence or a failure in the supply chain. This definition is a modified version of the Strand (1990) definition of a drug-related problem (DRP) ‘A DRP exists when a patient experiences or is likely to experience either a disease or symptom having an actual or suspected relationship with drug therapy’ [15]. This definition was agreed by a panel of experts (2 Professors of geriatric medicine, UK and Netherlands; 2 consultant geriatricians, UK; Professor of clinical pharmacy and therapeutics, UK; 2 clinical pharmacists specialising in geriatrics, UK).

### Baseline data collection

Baseline data will be collected by trained research nurses including demographic (e.g. age, gender, ethnicity), clinical (e.g. discharge diagnosis, co-morbidities, renal and hepatic function) and social indicators (e.g. care package received and living arrangements) using a form specifically designed to allow the data to be scanned into an electronic database for future analysis. Information relating to medication name, frequency, dose and use of compliance aids will be collected and coded according to the WHO-ATC code (http://www.whocc.no/atc_ddd_index/). In addition validated tools will be used to collect information relating to nutritional status (**M**alnutrition **U**niversal **S**creening **T**ool), physical function (Barthel Activities of Daily Living Index, Hand Grip strength), cognitive function (**A**bbreviated **M**ental **T**est **S**core) and depression and anxiety (**P**atient **H**ealth **Q**uestionnaire-**2**, **G**eneralised **A**nxiety **D**isorder scale-**2**). Some of these tool are routinely measured in elderly care wards in the UK and if this was not the case the research nurse would obtain the measurement. The hand grip strength of participants will be measured using the method described in the Southampton Protocol for Adult Grip strength Measurement using the JAMAR Hydraulic Hand Dynamometer [16]. The MUST score is routinely used on elderly care wards and is a five-step screening tool to identify adults, who are malnourished, at risk of malnutrition (undernutrition) or obese [17]. The Barthel ADL Index is a validated scale used to measure performance in activities in daily living (ADL) [18].

Data will be collected directly from the hospital records, following discussion with members of the care team and with patients and/or carers.

Following discharge the junior doctor from the discharging medical team will be asked to complete a questionnaire to determine their judgement of the likelihood of the patient experiencing MRH during the 8 week follow up period. This section of the data collection form is based on the National Patient Safety Agency (NPSA) Risk Model Matrix where the likelihood of an event is rated against the consequence [19]. The junior doctor will be asked to predict the likelihood that the patient will be readmitted or access healthcare in the community due to MRH in the ensuing 8 weeks post discharge (doubtful, possible, probable, definite) and will be asked to rate their confidence in this decision (a 6 point scale from ‘little or no confidence’ to ‘virtually certain’).

### Follow up

Eight weeks post discharge the research pharmacist at each site will conduct a telephone interview with the patient and/or carer using a standard questionnaire to determine whether the patient has experienced MRH. The patient/carer will be asked about their health service utilization over the preceding 8 weeks (GP visit, Out of Hours visit, hospital attendance/re-admission). The patient’s adherence to their medications will also be determined, and the patient will be asked whether they have recognized any unwanted reactions or effects from their medications. The research pharmacists will also review the GP patient records to determine whether the patient had experienced MRH and had, as a consequence, required additional health care support.

Any re-admitted patient will continue to be followed up for the eight week period after the original discharge date and telephone follow-up and GP record data will be collected as standard.

Patients who completed their eight week follow-up period and wish to participate in the study again, following a repeat admission, will be allowed re-enter the study. They will be allocated a new UPIN which will be linked to their first UPIN to allow for sub-analysis of patients who are re-admitted to hospital.

**Fig. 2:**
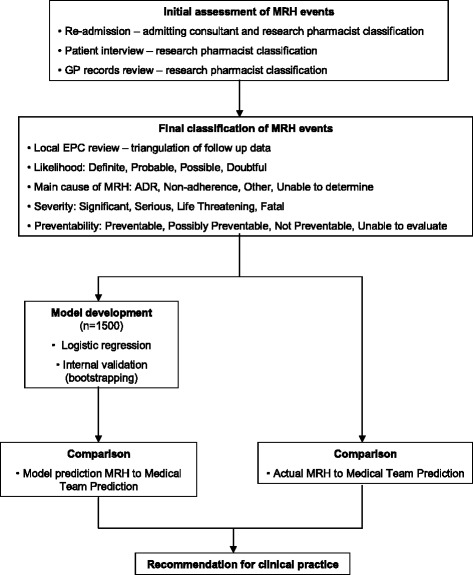
Clinical decision making flow chart. This flow chart provides a simple outline of the method which will be employed to perform an intial assessment at follow-up of whether medication harm has occurred based on patient interview, hospital re-admission and General Practice records. This will be followed by a final assessment of potential medication harm events, classified by likelihood, cause, severity and preventability of the event. The binary outcome of this assessment to determine the occurrence (or not) of medication harm will inform the logistic regression and risk prediction model development. The model will be internally validated and its ability to predict medicaiton harm will be compared to the ability of discharging clinicians to predict medication harm at the point of hospital discharge

Please see Fig. 2 for a flow-chart detailing this follow-up process.

### Decision making

- Key information required to support the research pharmacists (and consultant physicians where relevant) in determining the likelihood that the patient has experienced MRH include current medications and any recent changes to medication, assessment of patient’s adherence, history of presenting complaint and ADR profile of the prescribed medicines, relevant co-morbidities, and appropriate clinical observations and investigations. MRH will be categorised as doubtful, possible, probable, or definite.
- Where an ADR is suspected the Naranjo algorithm will be utilised to support the causality assessment [20]. The Naranjo algorithm rates the causality conservatively as many of questions are not relevant e.g. did the same reaction occur when placebo was administered? Therefore it will be used as a guide to ensure temporal association, previous reports of the reaction and other possible causes are all considered when determining MRH due to an ADR.
- For an assessment of patient adherence to their medications, the Morisky scale [21] will be used.
- Where MRH is unclear, or if the admitting consultant and pharmacist cannot agree for re-admissions, cases are presented by the research pharmacist to the local End Point Committee (EPC) for further discussion and decision making.
- If a patient was re-admitted during the 8-week follow up period, data pertaining to that re-admission will be collected. The likelihood that the re-admission was due to MRH will be assessed by the research pharmacist and the admitting consultant physician using a standardized approach which incorporates the outcome from the Naranjo algorithm and the Morisky scale (as outlined above) (Table 1).

**Table 1:** The process by which Medication-related harm outcomes are recorded

Question	Options
- 1. Do you think this patient has suffered medication related harm?	○ Definite, Probable, Possible, Doubtful
- 2. How confident are you in this judgement?	○ little or no confidence, slight to moderate confidence, <50 % confidence but a close call, >50 % confidence but a close call, strong confidence, virtually certain
- 3. If the patient has suffered medication related harm, what was the main cause?	○ ADR, non-adherence, other, unable to determine
- What medications were implicated?	Free text entered by research pharmacist
- What was the clinical event of the MRH?	Free text entered by research pharmacist
- 4. If the patient has suffered medication related harm, was it preventable?	○ Definitely, possibly, not preventable, not able to determine
5. If the patient has suffered medication related harm, what was the severity?	○ Fatal, life threatening, serious, significant

### Triangulation of outcomes

The outcomes recorded for each follow up stage (i.e. Patient telephone interview, GP records review, and re-admission where applicable) will be reviewed to determine the final outcome.

For each event the following will be recorded with the benefit of the complete follow-up by either the lead research pharmacist (JS), research fellow (NP), Professor of Clinical Pharmacy & Therapeutics (GD) or the Chief Investigator (KA):

1. Has the patient suffered an MRH? Definite; Probable; Possible; Doubtful
2. Was the MRP preventable? Definite; Possible; Not preventable
3. What was the severity? Fatal; Life threatening; Serious (hospitalisation); Serious (A&E); Serious (Community Care); Serious (Self-management); Significant
4. What was the main cause? ADR; Non-adherence; both
5. What was the event?
6. What was the drug(s)?

The total number of events and the healthcare utilization (re-admission, A&E attendance, access to out of hours services, GP or pharmacist) due to MRH will be recorded.

### Withdrawal arrangements

Any patient who wishes to withdraw from the study is free to do so at any point without giving any reason.

### Loss to follow up

Every effort will be made by the research pharmacist to trace participants lost to follow up. Hospital database, GP records, and contact with any named next of kin will be undertaken to determine whether the patient is still alive, their state of health at the follow up point, and if there are any new contact details.

### End of study

The study will end when the final participant has completed the 8- weeks follow up.

### Reporting of adverse events

Adverse medication-related events will be discussed by the local EPC as appropriate and the patient’s admitting physician or patient’s GP will be subsequently notified.

### Sample size

The sample size calculation was determined to achieve a sensitivity of 80 % with a 95 % confidence interval width of 5 % and based on a medication related problem prevalence rate of 30 %. The nomogram designed by Carley et al. [22], based on the work of Buderer [23], was used to determine the sample size of 1500 patients. A maximum of 50 % of the total study population may be recruited from one site to reduce the risk of an unrepresentative study population.

### Statistical analysis

Data collected for the potential risk variables will undergo univariate analysis to identify the variables significantly associated with binary outcome of MRH. All variables, significant and non-significant, will be reviewed by the expert panel for clinical relevance and consistency with the literature. This will be conducted without prior knowledge of the statistical relationships of the data to avoid introducing observer bias. It is recognised that variables deemed non-significant upon univariate analysis should not be removed automatically; rather their significance should be reviewed by experts in the field, especially if the data set is small or the prevalence is rare [24]. Significant variables prevalent in less than 5 % of the study population will be reviewed and potentially rejected if they are not considered to be representative of the population. Correlation between pre-determined variables with likely interaction e.g. number of co-morbidities and number of medicines will be assessed. The dichotomisation of data, that is categorising continuous data into two groups, will be avoided where possible [25]. The variables will undergo multivariable logistic regression analysis and be eliminated or retained in the model as indicated by a combination of clinical and statistical significance.

Internal validation of the RPM using bootstrapping will follow this.

Descriptive statistics will be applied to describe population characteristics, healthcare utilisation and to identify any significant differences between those who experienced MRH and those who did not. Odds ratios will be calculated to determine the odds of a specific medication group being associated with the need for patients to access unplanned support. Multivariable regression analysis will be applied to candidate variables to identify the variables which, when combined, produce the optimal RPM sensitivity and specificity. Model calibration and discrimination will be calculated using appropriate tests e.g. Hosmer-Lemeshow and Area Under the Receiver Operating Characteristic (AUROC) curve [25]. A comparison of the predictive power of the model to the doctors’ routine judgement will be conducted using appropriate statistical techniques.

## Data protection

### Participant identification, data archiving, and, storage

All patients will be given a unique study number to preserve confidentiality. This number will be entered into an appropriate database and will only be traceable to an individual patient by accessing the study form for the patient from a secure location at the Research and Development Department at each study centre. All electronic data will be password protected and only direct members of the research team will have access to the full set of electronic data generated within the study. Hard copies of all data collection forms will be stored at respective study sites for a maximum of 3 years after the study has ended.

### Ethical considerations

If during the data collection a concern is raised by the research nurse or the pharmacist, the issue will be discussed with the local Principal Investigator (PI), and advice will be given to notify the patient’s GP and their clinical teams.

### Protocol compliance and deviation/violation

Notification of violation to sponsor.

## Discussion

The impact of MRH in the community, and related incidence of hospital admission has significant implications for the health and quality of life of elderly people, as well as the economic burden of avoidable primary and secondary healthcare service utilisation. Currently there is no standardized method for identifying older people at high risk of MRH upon discharge and thus there is no systematic approach to guide appropriate monitoring and intervention as needed in the community. Existing RPMs in the context of ADR in older people that have been developed for use within the hospital setting, and have shown at best a moderate performance (AUROC 0.623 to 0.73) and only two models have been externally validated [8, 9]. Therefore these prediction models are not in widespread clinical application. The aim of this study is to develop a clinically valuable and practical RPM that can identify elderly people at high risk of MRH following discharge from hospital.

It is hoped that the RPM developed through this study will alert discharging doctors of patients at high risk for whom an early medication review, follow-up and/or adjustment of discharge medications may be indicated. A wide range of social factors (e.g. Activities of Daily Living, Accommodation status) and measurements for patient frailty (e.g. handgrip strength) are being measured in patients recruited for this study, which might support a model of higher predictive value than other tools developed in the context of MRH. Frailty has not been previously been explored as potentially important variable within a RPM for medication problems. Frailty is a common geriatric syndrome that embodies a decline in health and function associated with ageing. It is characterized by a loss of functional homeostasis such that a minor insult can result in catastrophic consequences for the individual.

A further special and key feature of this study is the comparison of the RPM that will be developed with routine risk prediction by junior doctors upon discharge of older people. This will help to determine whether the RPM developed is superior to current best practice.

It is acknowledged that asking the junior doctors to prospectively review patients’ risk in this way could affect their future behavior as described by the ‘Hawthorne Effect’. However, any learning effect is likely to be limited by the frequent rotation of junior doctors. Should this, however, influence the outcome of the study in such a way that raises awareness of high risk patients and results in a safer clinical judgment then this, in itself, would be a positive outcome.

### Study progress

Stage I of the study is complete, and we encourage readers to refer to the Stevenson et al. [26] systematic review of the existing RPMs to predict ADR in elderly people.

Stage II of the study is currently ongoing.

## Additional file

Additional file 1: Table S2. Candidate Predictor Variables. (DOCX 59 kb)

## Abbreviations

PRIME: Prospective (P) study to develop a model to stratify the risk (RI) of medication (M) related harm in hospitalized elderly (E)
MRH: Medication-related Hharm
ADR: Adverse drug reaction
RPM: Risk prediction model
EPC: End point committee
