# Supplementary material for: Protocol for a Prospective (P) study to develop a model to stratify the risk (RI) of medication (M) related harm in hospitalized elderly (E) patients in the UK (The PRIME study)
Source: BMC Geriatr. 2016 Jan 19;16:22. doi: 10.1186/s12877-016-0191-8 (PMC4719738; doi:10.1186/s12877-016-0191-8)
Supplement: Additional file 1: Table S2. — Candidate Predictor Variables. (DOCX 59 kb) [file 12877_2016_191_MOESM1_ESM.docx]

**Additional file 1: Table S2: Potential Candidate Predictor Variables**

| **Risk Variable** | **Level of importance (✓)** | | | **Point of collection (✓)** | | | | | **Source of data** | | **Method of collection** | | **Existing measure/scales** | | **Included in existing risk prediction tool** | **Include** | **Comment** |  |
| --- | --- | --- | --- | --- | --- | --- | --- | --- | --- | --- | --- | --- | --- | --- | --- | --- | --- | --- |
|  | **Essential** | **Desirable** | **Not required** | **Adm** | **D/C** | | **Other** | | **London** | **Brighton** | **London** | **Brighton** | **London** | **Brighton** |  | **(✓/🗶)** |  |  |
| **Medications** |  | | | | | | | | | | | | | | | | |  |
| Prescription Only Medications (POMs) |  |  |  |  |  | |  | |  |  |  |  |  |  |  |  |  |  |
| Over the Counter Medications (OTC) |  |  |  |  |  | |  | |  |  |  |  |  |  |  |  |  |  |
| When required medications (PRNs) |  |  |  |  |  | |  | |  |  |  |  |  |  |  |  |  |  |
| Once only medications (STAT) |  |  |  |  |  | |  | |  |  |  |  |  |  |  |  |  |  |
| Fluids |  |  |  |  |  | |  | |  |  |  |  |  |  |  |  |  |  |
| Blood products |  |  |  |  |  | |  | |  |  |  |  |  |  |  |  |  |  |
| Medication Name |  |  |  |  |  | |  | |  |  |  |  |  |  |  |  |  |  |
| Dose |  |  |  |  |  | |  | |  |  |  |  |  |  |  |  |  |  |
| Frequency |  |  |  |  |  | |  | |  |  |  |  |  |  |  |  |  |  |
| Number of doses per day |  |  |  |  |  | |  | |  |  |  |  |  |  |  |  |  |  |
| Route of admin |  |  |  |  |  | |  | |  |  |  |  |  |  |  |  |  |  |
| Duration (Acute/Chronic) |  |  |  |  |  | |  | |  |  |  |  |  |  |  |  |  |  |
| Change in medication |  |  |  |  |  | |  | |  |  |  |  |  |  |  |  |  |  |
| Medication Classification |  |  |  |  |  | |  | |  |  |  |  |  |  |  |  |  |  |
| Total number of medications |  |  |  |  |  | |  | |  |  |  |  |  |  |  |  |  |  |
| Use of compliance aid |  |  |  |  |  | |  | |  |  |  |  |  |  |  |  |  |  |
| Regular community pharmacist |  |  |  |  |  | |  | |  |  |  |  |  |  |  |  |  |  |
| Who administers meds |  |  |  |  |  | |  | |  |  |  |  |  |  |  |  |  |  |
| Patient/carer knowledge of the medication |  |  |  |  |  | |  | |  |  |  |  |  |  |  |  |  |  |
| Problems with medications e.g missed doses, side effects, running out |  |  |  |  |  | |  | |  |  |  |  |  |  |  |  |  |  |
| Patient thinks drug was responsible for admission |  |  |  |  |  | |  | |  |  |  |  |  |  |  |  |  |  |
| Previous ADR |  |  |  |  |  | |  | |  |  |  |  |  |  |  |  |  |  |
| Appropriate monitoring of medications e.g. warfarin at anticoag clinic |  |  |  |  |  | |  | |  |  |  |  |  |  |  |  |  |  |
| Number of prescribers (i.e. cross specialities) |  |  |  |  |  | |  | |  |  |  |  |  |  |  |  |  |  |
| **Social** |  | | | | | | | | | | | | | | | | |  |
| Marital status |  |  |  |  | |  | |  |  |  |  |  |  |  |  |  |  |  |
| Living arrangements (i.e. alone, with spouse, with family, RH, NH) |  |  |  |  | |  | |  |  |  |  |  |  |  |  |  |  |  |
| Smoking status |  |  |  |  | |  | |  |  |  |  |  |  |  |  |  |  |  |
| Alcohol consumption |  |  |  |  | |  | |  |  |  |  |  |  |  |  |  |  |  |
| Home nursing care |  |  |  |  | |  | |  |  |  |  |  |  |  |  |  |  |  |
| Monthly income |  |  |  |  | |  | |  |  |  |  |  |  |  |  |  |  |  |
| Volunteer work |  |  |  |  | |  | |  |  |  |  |  |  |  |  |  |  |  |
| Attendence at daycare centre |  |  |  |  | |  | |  |  |  |  |  |  |  |  |  |  |  |
| Number of times patient able to leave the house in the past 3 months as a result of their own efforts |  |  |  |  | |  | |  |  |  |  |  |  |  |  |  |  |  |
| Education (primary, secondary, tertiary) |  |  |  |  | |  | |  |  |  |  |  |  |  |  |  |  |  |
| ADL - need for assistance with one or more tasks: eating, dressing bathing transferring, toileting |  |  |  |  | |  | |  |  |  |  |  |  |  |  |  |  |  |
| Family support |  |  |  |  | |  | |  |  |  |  |  |  |  |  |  |  |  |
| Care package |  |  |  |  | |  | |  |  |  |  |  |  |  |  |  |  |  |
| **Biochemistry** | | | | | | | | | | | | | | | | | |  |
| Hb |  |  |  |  |  | | |  |  |  |  |  |  |  |  |  |  |  |
| RBC |  |  |  |  |  | | |  |  |  |  |  |  |  |  |  |  |  |
| PCV |  |  |  |  |  | | |  |  |  |  |  |  |  |  |  |  |  |
| MCV |  |  |  |  |  | | |  |  |  |  |  |  |  |  |  |  |  |
| MCH |  |  |  |  |  | | |  |  |  |  |  |  |  |  |  |  |  |
| MCHC |  |  |  |  |  | | |  |  |  |  |  |  |  |  |  |  |  |
| RDW |  |  |  |  |  | | |  |  |  |  |  |  |  |  |  |  |  |
| WCC |  |  |  |  |  | | |  |  |  |  |  |  |  |  |  |  |  |
| Neutrophils |  |  |  |  |  | | |  |  |  |  |  |  |  |  |  |  |  |
| Lymphocytes |  |  |  |  |  | | |  |  |  |  |  |  |  |  |  |  |  |
| Monocytes |  |  |  |  |  | | |  |  |  |  |  |  |  |  |  |  |  |
| Eosinophils |  |  |  |  |  | | |  |  |  |  |  |  |  |  |  |  |  |
| Basophils |  |  |  |  |  | | |  |  |  |  |  |  |  |  |  |  |  |
| Platelets |  |  |  |  |  | | |  |  |  |  |  |  |  |  |  |  |  |
| MPV |  |  |  |  |  | | |  |  |  |  |  |  |  |  |  |  |  |
| Troponin |  |  |  |  |  | | |  |  |  |  |  |  |  |  |  |  |  |
| CRP |  |  |  |  |  | | |  |  |  |  |  |  |  |  |  |  |  |
| Creatinine |  |  |  |  |  | | |  |  |  |  |  |  |  |  |  |  |  |
| Urea |  |  |  |  |  | | |  |  |  |  |  |  |  |  |  |  |  |
| Potassium |  |  |  |  |  | | |  |  |  |  |  |  |  |  |  |  |  |
| Sodium |  |  |  |  |  | | |  |  |  |  |  |  |  |  |  |  |  |
| eGFR |  |  |  |  |  | | |  |  |  |  |  |  |  |  |  |  |  |
| ALT |  |  |  |  |  | | |  |  |  |  |  |  |  |  |  |  |  |
| AST |  |  |  |  |  | | |  |  |  |  |  |  |  |  |  |  |  |
| ALP |  |  |  |  |  | | |  |  |  |  |  |  |  |  |  |  |  |
| TBIL |  |  |  |  |  | | |  |  |  |  |  |  |  |  |  |  |  |
| GGT |  |  |  |  |  | | |  |  |  |  |  |  |  |  |  |  |  |
| Albumin |  |  |  |  |  | | |  |  |  |  |  |  |  |  |  |  |  |
| INR |  |  |  |  |  | | |  |  |  |  |  |  |  |  |  |  |  |
| APTT |  |  |  |  |  | | |  |  |  |  |  |  |  |  |  |  |  |
| Vit B12 |  |  |  |  |  | | |  |  |  |  |  |  |  |  |  |  |  |
| Folate |  |  |  |  |  | | |  |  |  |  |  |  |  |  |  |  |  |
| Iron |  |  |  |  |  | | |  |  |  |  |  |  |  |  |  |  |  |
| TC |  |  |  |  |  | | |  |  |  |  |  |  |  |  |  |  |  |
| LDL |  |  |  |  |  | | |  |  |  |  |  |  |  |  |  |  |  |
| HDL |  |  |  |  |  | | |  |  |  |  |  |  |  |  |  |  |  |
| Blood glucose |  |  |  |  |  | | |  |  |  |  |  |  |  |  |  |  |  |
| HbA1C |  |  |  |  |  | | |  |  |  |  |  |  |  |  |  |  |  |
| Measured serum drug concentration |  |  |  |  |  | | |  |  |  |  |  |  |  |  |  |  |  |
| **Medical** | | | | | | | | | | | | | | | | | |  |
| Presenting complaint |  |  |  |  |  | | |  |  |  |  |  |  |  |  |  |  |  |
| Diagnosis on admission |  |  |  |  |  | | |  |  |  |  |  |  |  |  |  |  |  |
| Diagnosis on discharge |  |  |  |  |  | | |  |  |  |  |  |  |  |  |  |  |  |
| Cause of hospitalisation (ADE v non-ADE) |  |  |  |  |  | | |  |  |  |  |  |  |  |  |  |  |  |
| Number of acute medical problems |  |  |  |  |  | | |  |  |  |  |  |  |  |  |  |  |  |
| Number of past medical problems |  |  |  |  |  | | |  |  |  |  |  |  |  |  |  |  |  |
| Co-morbidities |  |  |  |  |  | | |  |  |  |  |  |  |  |  |  |  |  |
| Number of co-morbidities |  |  |  |  |  | | |  |  |  |  |  |  |  |  |  |  |  |
| Number of hospital admissions in past 12 months |  |  |  |  |  | | |  |  |  |  |  |  |  |  |  |  |  |
| Number of different visits to clinic in past 12 months |  |  |  |  |  | | |  |  |  |  |  |  |  |  |  |  |  |
| Number of different visits to hospital (outpatients) in last 12 months |  |  |  |  |  | | |  |  |  |  |  |  |  |  |  |  |  |
| Last doctor attended (GP or hospital doctor) |  |  |  |  |  | | |  |  |  |  |  |  |  |  |  |  |  |
| Number of GP visits in past year |  |  |  |  |  | | |  |  |  |  |  |  |  |  |  |  |  |
| Length of hospital stay |  |  |  |  |  | | |  |  |  |  |  |  |  |  |  |  |  |
| Self-reported health in last year |  |  |  |  |  | | |  |  |  |  |  |  |  |  |  |  |  |
| Weight/height ratio |  |  |  |  |  | | |  |  |  |  |  |  |  |  |  |  |  |
| Nutrition |  |  |  |  |  | | |  |  |  |  |  |  |  |  |  |  |  |
| Method of feeding |  |  |  |  |  | | |  |  |  |  |  |  |  |  |  |  |  |
| Recent nausea/vomiting/diarrhoea |  |  |  |  |  | | |  |  |  |  |  |  |  |  |  |  |  |
| Temperature |  |  |  |  |  | | |  |  |  |  |  |  |  |  |  |  |  |
| Pulse |  |  |  |  |  | | |  |  |  |  |  |  |  |  |  |  |  |
| Blood pressure |  |  |  |  |  | | |  |  |  |  |  |  |  |  |  |  |  |
| Hearing eyesight |  |  |  |  |  | | |  |  |  |  |  |  |  |  |  |  |  |
| Mental/cognitive status |  |  |  |  |  | | |  |  |  |  |  |  |  |  |  |  |  |
| Renal failure |  |  |  |  |  | | |  |  |  |  |  |  |  |  |  |  |  |
| Indicators of physical function |  |  |  |  |  | | |  |  |  |  |  |  |  |  |  |  |  |
| Falls |  |  |  |  |  | | |  |  |  |  |  |  |  |  |  |  |  |
| Ischaemic heart disease |  |  |  |  |  | | |  |  |  |  |  |  |  |  |  |  |  |
| Diabetes |  |  |  |  |  | | |  |  |  |  |  |  |  |  |  |  |  |
| Infections |  |  |  |  |  | | |  |  |  |  |  |  |  |  |  |  |  |
| Acute stroke |  |  |  |  |  | | |  |  |  |  |  |  |  |  |  |  |  |
| Liver disease |  |  |  |  |  | | |  |  |  |  |  |  |  |  |  |  |  |
| Congestive cardiac failure |  |  |  |  |  | | |  |  |  |  |  |  |  |  |  |  |  |
| Angina |  |  |  |  |  | | |  |  |  |  |  |  |  |  |  |  |  |
| COPD |  |  |  |  |  | | |  |  |  |  |  |  |  |  |  |  |  |
| Hyperlipidaemia |  |  |  |  |  | | |  |  |  |  |  |  |  |  |  |  |  |
| **Other** | | | | | | | | | | | | | | | | | |  |
|  | | | | | | | | | | | | | | | | | |  |
| Adm: Admission; D/C: Discharge; ADR: Adverse Drug Reaction; RH: Residential Home; NH: Nursing Home; ADL: Activity of Daily Living; ADE: Adverse Drug Event | | | | | | | | | | | | | | | | | |  |
